# Awareness of age-related changes in Norwegian individuals 50+. Short form questionnaire validation

**DOI:** 10.3389/fpsyt.2022.929249

**Published:** 2022-11-10

**Authors:** Ingelin Testad, Anastasia Ushakova, Jon Arild Aakre, Serena Sabatini, Martha Therese Gjestsen

**Affiliations:** ^1^Centre for Age-Related Medicine - SESAM, Stavanger University Hospital, Stavanger, Norway; ^2^College of Medicine and Health, University of Exeter, Exeter, United Kingdom; ^3^Faculty of Biomedical Sciences, University of Italian Switzerland, Lugano, Switzerland

**Keywords:** awareness of age-related changes, healthy ageing, PROTECT Norge, online assessment, questionnaire validation, older adults

## Abstract

**Background:**

A questionnaire assessing awareness of positive and negative age-related changes (AARC gains and losses) was developed in the US and Germany, and validated for the UK and Brazilian populations. In this study, we validated the short-form measure (AARC-10 SF) in the Norwegian population aged 50 and over. In addition, the relationship between cognitive variables and AARC was examined.

**Methods:**

Cross-sectional analyses of data from 1,510 participants in the ongoing online PROTECT Norge study were used to explore and confirm the two-factor structure of AARC gains and AARC losses; reliability; measurement invariance across different population groups defined by sex, education level, employment, and in middle age, early old age, and advanced old age. We explored the relationship between AARC and demographic variables (defined in the same way as the population groups).

**Results:**

We confirmed the two-factor structure (gains and losses) of the Norwegian translation of the AARC-10 SF. We did not find mutual correlations between related items in gains and losses, except for the physical health item from the gains dimension, which was positively correlated with all items of the losses dimension. Age, sex, marital status, employment, and university education predicted AARC gains and losses.

**Conclusion:**

The Norwegian translation of the AARC-10 SF captures individuals’ positive and negative self-perceptions of age-related changes in their mental, physical, and cognitive health.

## Introduction

Globally, we expect to see a considerable growth in the aging population over the next decade, and such a demographic shift will strain health and social systems as we know them today (1). Healthy aging is defined by WHO as a “continuous process of optimizing opportunities to maintain and improve physical and mental health, independence, and quality of life throughout the life course” [(2); p. 2]. The healthy aging concept is proposed as a counteracting strategy on a societal level to ease such burdens. On an individual level, how people reflect on their own aging, affects their health and wellbeing (3). The concept of awareness of age-related change (AARC) refers to “a person’s state of awareness that his or her behavior, level of performance, or way of experiencing life has changed as a consequence of having grown older” [(3); p. 342]. Higher levels of awareness of positive age-related changes (AARC gains) and lower levels of awareness of negative age-related changes (AARC losses) are informative of better concurrent cognitive, physical, and mental health among middle-aged and older individuals (4–6). More AARC gains and fewer AARC losses also predict better future physical and mental health and even less mortality risk (7–9). This may be due to people perceiving more AARC gains and fewer AARC losses as being more engaged in those behaviors that foster health maintenance in middle and older age. Indeed, those with more AARC gains and fewer AARC losses are more likely to adapt to age-related challenges (10, 11) and engage in physical exercise and leisure activities (12, 13). In sum, asking individuals about the gains and losses they experience in several domains of their lives may help to identify those who are struggling the most while getting older. Among available versions of the AARC questionnaire, the AARC-10 SF (14) is the most currently used; it has been and is currently being administered as part of several cohort studies of aging, including the UK version of the PROTECT study, the German Socio-Economic Panel—Innovation Sample, the National Study of Daily Experiences (a sub-study of the Midlife in the United States study), and the Longitudinal Aging Study Amsterdam (LASA) in the Netherlands. The AARC-10 SF contains 10 items, 5 assessing AARC gains and 5 assessing AARC losses in different AARC behavioral domains: lifestyle/engagement, cognitive functioning, health and physical functioning, socio-cognitive and socio-emotional functioning and interpersonal relationships. The associations of demographic characteristics with levels of AARC gains and AARC losses seem to vary across cohorts and countries (15–18). Hence, it is important to have this validated for the Norwegian study cohort to enable cross-country comparison. This will increase the knowledge base of awareness of age-related change, which in turn can contribute to facilitate active and healthy aging.

It is crucial to explore whether the AARC concept is interpreted consistently in the same way across different populations. We have therefore assessed the measurement invariance, applying the socio-demographic characteristics age, sex, education level, marital status and work status as predictors of AARC gains and AARC losses. We have done so as previous research has demonstrated that they may be related to levels of mental, physical, and cognitive health in older age (19). An individual’s chronological age, as opposed to felt age, is a predictor of health decline (20). AARC is assessed in direct reference to one’s increasing age, and research has shown associations between higher levels of AARC losses and increased age (10). Increasing age is also associated with a more limited future time perspective (16). Previous evidence suggests that contrary to women in the US, German and UK women experience more AARC gains than men, and only in the UK do women report fewer AARC losses than men (18, 19). This supports the notion that there are generally differences in levels of gains and losses between men and women (15, 17). An increasing body of research has explored the associations between education and health (21, 22). It is quite established that the health effects of education are creating better overall self-awareness on personal health and making healthcare more accessible (23). In the Norwegian population, there has been a shift in the educational level of an aging population during the last decades; as the younger cohorts enter old age, they have a higher education (24). Another trend is that fewer older adults are expected to live alone, and this is supported by analyses of gender differences in the older population, which have been reduced over the last decade. There is a well-established link between employment status and health, with unemployment being associated with poorer health (25), but these variables (marital status and employment status) are still novel in relation to AARC gains and losses (18).

AARC might be associated with cognitive functioning, and the AARC-10 SF includes two items assessing AARC gains and AARC losses, respectively, in the cognitive functioning domain (18). Although the subjective perception of cognition is unspecific and is related to numerous factors such as general aging, personality traits, mental health issues, neurologic and medical disorders, substance use, and medication, in addition to be affected by the individual cultural background (26), the association between cognitive complaints and cognitive performance is well-reported in the empirical literature among the five AARC behavioral domains (5). Thus, the cognitive functioning domain is potentially useful for detecting early stages of cognitive decline.

This study uses a sample of cognitively healthy people aged 50 and over, living in Norway, and included in the PROTECT-Norge study, which is tailor-made to enable large-scale longitudinal data collection online and to deliver affordable clinical trials on aging individuals.

We aim to (1) confirm the two-factor structure (one factor for each of AARC gains and AARC losses) and the internal consistency of the Norwegian translation of the AARC-10 SF; (2) explore whether the factors AARC gains and AARC losses and each item of the AARC-10 SF are consistently interpreted in the same way (i.e., measurement invariance) across different population groups defined by sex, education level, employment, and age groups i.e., middle-aged (aged 50 to 65), early-old (aged 66–75), old-old (aged 76 and over), and (3), explore whether demographic characteristics, such as age, sex, education level, marital status, and work status predict levels of AARC gains and AARC losses.

## Materials and methods

### Study design and participants

Cross-sectional analysis of data from the ongoing online PROTECT Norge study,^1^ included individuals aged 50 or above, living in Norway and with access to a computer or tablet. Having an established diagnosis of dementia was an exclusion criterion for the study. In the cognitive test instruction, the participants were reminded not to use a smart phone when completing the cognitive assessments for data quality purposes. The PROTECT Norge study has been publicized in the national media, but mainly, targeted recruitment has been and is currently still taking place on the internet via social media platforms where potential participants are signposted to the study website to conduct a self-eligibility check, before continuing the registration and subsequent consent process.

### Measures

After being enrolled in the study, participants were asked to complete a series of online questionnaires regarding demographic, medical, and lifestyle information.

The study is ongoing, so participants are still enrolling; thus, the AARC-10 SF questionnaire is optional for the study participants and is filled out upon entering the study (baseline) and the subsequent annual follow-ups. Fifty-seven percentage of all participants have completed this questionnaire at baseline—and it forms the basis of our analyses. The 10 questions all begin with the words “With my increasing age, I realize that….” All questions are presented in Table 2, as well as their interpretation in terms of underlying functioning and attribution to the gains- and losses- domains. All items are rated on a five-point Likert scale ranging from 1 (not at all) to 5 (very much). AARC gains- and losses- scores were obtained by summing the five items from the respective domains. Higher scores indicate higher levels of AARC gains and AARC losses, respectively.

Guided by Beaton et al. (27), the process of translating the AARC-10 SF from English to Norwegian, included two separate forward translations into Norwegian by native speakers, fluent in both languages. They later met, discussed and agreed upon a consensus version. A translator with English as native language and a second translator with Norwegian as first language, both bilingual, provided two independent back translations of the consensus version. The audit trail from each stage of the translation process thus far, and all versions of the translated measure, both forward and back translations, were reviewed by an expert committee consisting of translators from all stages of the translation process and members of the PROTECT Norge working group. The expert committee agreed on a final preliminary Norwegian version of the AARC 10 SF. This version was then meticulously tested in an electronic format, on the PROTECT Norge platform, by both end users and user representatives, members of the PROTECT Norge working group and other researchers, up to a total of more than 30 individuals.

Cognitive functioning was assessed through a self-administered online cognitive test battery (28). Participants were encouraged to complete the battery in triplicate within 1 week, with at least 12 h separating each try, although triplicate testing was not mandatory. The PROTECT cognitive test battery includes six tests: Self-Ordered Search task assessing spatial working memory, Grammatical Reasoning task assessing verbal reasoning, Paired Associate Learning task assessing visual episodic memory, Digit Span task assessing verbal working memory and Trail-making B task and Switching Stroop task, assessing visual attention and task switching. By subtracting the number of errors from the number of correct answers in each test, a total score was obtained. The average score of all available (maximum 3) attempts was used for the analysis.

### Statistical analyses

Categorical variables were presented using count and percentage; age variable was presented using median and interquartile range (IQR). Questionnaire items were described using mean and standard deviation (SD). Cronbach’s alpha (α*^C^*) was used to quantify reliability for the gains and losses subscales of the AARC. We considered α values over 0.7 as acceptable (29). Pearson correlation (*R*) and test were used to evaluate linear associations between the items; Bonferroni correction for multiple testing was applied for correlation analysis.

Confirmatory factor analysis (CFA) was used to confirm the two-factor structure of the Norwegian translation of the AARC-10 SF (14). We tested if the five items assessing gains and the five items assessing losses were related to the hypothesized underlying factors. The two factors, AARC gains and AARC losses were assumed to be correlated. Residual correlations were assumed for the pairs of gains and losses items of the same AARC behavioral domain. Goodness-of-fit was evaluated using the Comparative Fit index (CFI), the Tucker-Lewis index (TLI), the Root Mean Square Error of Approximation (RMSEA) with the 90% confidence interval (CI), and the Standardized Root Mean Square Residual (SRMR). Cut-off values for acceptable model fit were CFI and TLI > 0.9 (30), RMSEA < 0.08 (90% CI: between 0 and 0.08) (31, 32) and SRMR < 0.08 (30). To improve the model fit and better understand the structure of the data, we applied the modification indices method (32). In short, the modification indices method considers all single additions of cross-loadings and residual correlation elements into the CFA model and evaluates the corresponding change of the χ^2^-statistic of the model fit; it is an exploratory method for identifying the strongest candidates for additional cross-loadings.

To explore measurement invariance, we used multiple-group CFA. For each grouping variable, we considered three CFA models with the same two-factor structure as the AARC-10 SF:

1. Model 1: Configural invariance (no constraints on parameters).
2. Model 2: Metric invariance (factor loadings are constrained to be identical across subgroups).
3. Model 3: Strong invariance (factor loadings and item intercepts are constrained to be identical across subgroups).

We concluded that a more constrained model had a worse fit compared to a less constrained model when the difference in CFI (ΔCFI) was larger than -0.01, the difference in RMSEA (ΔRMSEA) was larger than 0.015, and the difference in SRMR (ΔSRMR) was larger than 0.01 (33).

To explore whether age, sex, marital status, employment status, and university education explains variability in levels of AARC gains and/or AARC losses, we fitted simple linear regression models, with AARC gains and AARC losses serving as the outcome variables. In order to control for potential confounding effects, we also conducted multiple regressions with AARC gains or AARC losses as the outcome and demographic variables (i.e., age, sex, marital status, employment status, and university education) included as predictors.

All data analyses were performed using R Project for Statistical Computing version 4.1.2. Confirmatory factor analyses were performed using R package **lavaan** version 0.6-9.

## Results

Demographic characteristics of the data are presented in Table 1. Most of the participants were female (79.7%). The age range was between 50 and 86 years old; 28.6% of the participants were living alone and 50.8% had either a full-time or a part-time job.

**TABLE 1:** Demographic characteristics of study participants (*N* = 1,510).

Characteristic	Statistic
Age, median (IQR)	63.2 (57.1; 69.1)
Female, n (%)	1,204 (79.7)
Ethnicity, n (%)	
Western European/North American/Oceania	1,465 (97.0)
Other ethnic origin	45 (3.0)
Marital status, n (%)	
Married/civil partnership/co-habiting	1,074 (71.4)
Widowed/separated/divorced/single	430 (28.6)
University education, n (%)	1,156 (76.6)
Current employment, n (%)	764 (50.8)

**Cognitive tests**	

Digit span, median (IQR)	6.3 (5.0; 7.5)
Paired associate learning, median (IQR)	4.0 (3.0; 4.0)
Grammatical reasoning, median (IQR)	25.0 (18.0; 32.0)
Self-ordered search, median (IQR)	7.0 (6.0; 8.0)
Switching stroop test, median (IQR)	34.0 (24.0; 44.0)
Trail-making B, median (IQR)	64,162 (53,914; 79,445)

To examine the selectivity of the group of participants who completed the AARC-10 SF, we compared them with the group who did not complete it by their baseline characteristics (as in Table 1). There was a difference between the ethnical groups (*P*-value < 0.0001), with 41.5% of the participants of western origin not completing the AARC questionnaire, vs. 61.5% non-compliance in the group with other ethnicities. Completion rates also differed between the groups with and without a co-habitant (*P*-value < 0.001), with 48.6% in the group living alone vs. 39.8% in the group of people living with a partner.

### Item characteristics and internal consistency

Cronbach’s alpha (α*^C^*) demonstrated acceptable internal consistency of 0.74 for AARC gains and 0.81 for AARC losses. Items “… *I have a better sense of what is important for me*” and “*I feel more dependent on the help of others*” got the highest and the lowest mean scores, respectively. Variations of the scores were similar among all questions (all SDs between 0.8 and 1.1, Table 2).

**TABLE 2:** Item characteristics.

Item	With my increasing age, I realize that…	Mean	SD	Item-total correlation
PHYS+	… *I pay more attention to my health*	2.0	0.9	0.56
COG+	… *I have more experience and knowledge to evaluate things and people*	2.3	0.9	0.50
INT+	… *I appreciate relationship and people much more*	2.5	1.1	0.54
SCSE+	… *I have a better sense of what is important for me*	2.7	0.9	0.60
LIFE+	… *I have more freedom to live my days the way I want*	2.5	1.0	0.48
PHYS−	… *I have less energy*	1.9	1.0	0.62
COG−	… *my mental capacity is declining*	1.2	0.9	0.57
INT−	… *I feel more dependent on the help of others*	0.6	0.8	0.57
SCSE−	… *I find it harder to motivate myself*	0.7	0.8	0.48
LIFE−	… *I have to limit my activities*	0.9	0.9	0.55

AARC domain abbreviations: PHY, health and physical functioning; COG, cognitive functioning; INT, interpersonal relations; SCSE, social-cognitive and social-emotional functioning; LIFE, lifestyle and engagement. “+” stands for positive domains, and “−” stands for negative domains.

The correlation analysis revealed an unusual pattern among the participants: there was a modest but statistically significant correlation between the question “… *I pay more attention to my health*,” which assesses gains in the physical health domains, and all the items attributed to AARC losses (*R* = 0.2–0.3, Table 3). In addition, the mutual correlations between related items in the gains and losses domains included in the definition of the two-factor AARC-10 SF model (34) were not observed in this data set.

**TABLE 3:** Correlations between items.

	… *I pay more attention to my health*	… *I have more experience and* knowledge to evaluate things and people	… *I appreciate relationship* and people much more	… *I have a better sense of what* is important for me	… *I have more freedom to live my days* the way I want	… *I have less energy*	… *my mental capacity is declining*	… *I feel more dependent on the* help of others	… *I find it harder to motivate myself*	… *I have to limit my activities*
**… *I pay more attention to my health***		0.3	0.2	0.3	0.2	0.2	0.2	0.2	0.2	0.2
**… *I have more experience and knowledge to evaluate things and people***	0.3		0.3	0.5	0.4					
**… *I appreciate relationship and people much more***	0.2	0.3		0.6	0.4			0.1		
**… *I have a better sense of what is important for me***	0.3	0.5	0.6		0.5	0.1		0.1		
**… *I have more freedom to live my days the way I want***	0.2	0.4	0.4	0.5						
**… *I have less energy***	0.2			0.1			0.5	0.4	0.4	0.6
**… *my mental capacity is declining***	0.2					0.5		0.5	0.4	0.4
**… *I feel more dependent on the help of others***	0.2		0.1	0.1		0.4	0.5		0.4	0.5
**… *I find it harder to motivate myself***	0.2					0.4	0.4	0.4		0.4
**… *I have to limit my activities***	0.2					0.6	0.4	0.5	0.4	

Only statistically significant after correction for multiple testing are presented.

### Confirmatory factor analysis

**FIGURE 1:**
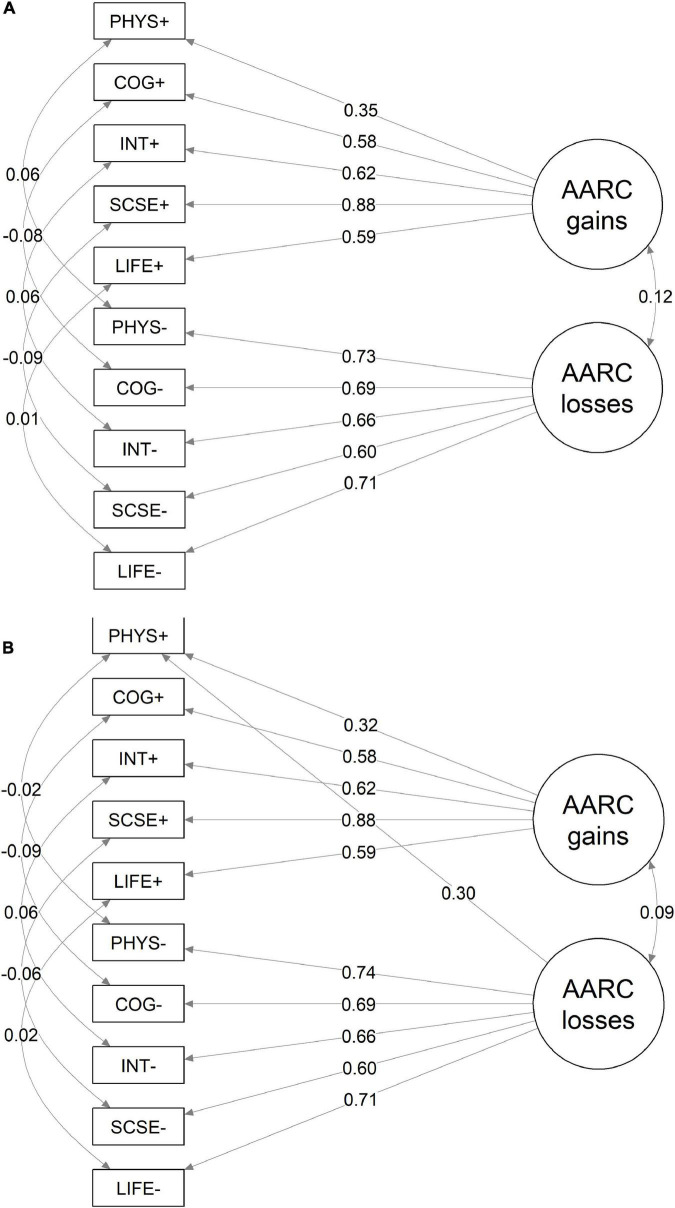
Parameter estimates of two-factor model of the AARC-10 SF **(A)** and the modified model **(B)**. Standardized coefficients and estimated residual correlations are presented. AARC Domain abbreviations: PHY, Health and physical functioning; COG, Cognitive functioning; INT, Interpersonal relations; SCSE, Social-cognitive and social-emotional functioning; LIFE, Lifestyle and engagement. “+” stands for positive domains, “–” stands for negative domains.

Goodness-of-fit indices indicated an acceptable model fit using TLI and CFI criteria (0.92 and 0.95, respectively). RMSEA = 0.07 (90%CI 0.06–0.08) and SRMR = 0.06 were higher than their conventional cut-off values of 0.05 (35), but lower than the less restrictive cut-off of 0.08 as previously suggested. A graphical presentation of the estimates is presented in Figure 1.

To further uncover a possible hidden pattern inside the data, we applied the modification indices method. A strong evidence of cross-loading of the question “… *I pay more attention to my health*” on the losses factor was established: the modification index was 113.0, and the χ^2^-square statistic of the model fit improved from 247.5 to 130.6.

Inclusion of this cross-loading improved dramatically the model fit [TLI = 0.96, CFI = 0.98, RMSEA = 0.05, (90% CI 0.04–0.06) SRMR = 0.03].

The interpretation of this can be; that in a Norwegian population, the “*I pay more attention to my health*” item can be counted in both gains and losses scales as people who are in poorer health and have health conditions may also be encouraged to “pay more attention” to their health. We emphasize the similarity of the standardized estimates for the two models for all domains except for the domain of health and physical functioning (Figure 1).

All further analyses were reported for the original AARC-10 SF two-factor model and repeated for the model modified by including the cross-loading of “… *I pay more attention to my health*” to losses domain (which is referred to as the model with cross-loading). For each analysis, it was ensured that the results remained unchanged, and any differences in the results were reported.

### Measurement invariance

Measurement invariance for the classes defined by sex, marital status and education group were established for the main model (Table 4), since all the differences between the less- and the more constrained models were within the pre-defined limits for all the goodness-of-fit measures (|△CFI| < 0.01, △RMSEA < 0.015, △SRMR < 0.03). For the age groups and the employment classes, △RMSEA and △SRMR were within the limits, while △CFI exceeded the cut-off for strong invariance slightly. However, since all the goodness-of-fit measures (including CFI) still fell within the pre-defined range for acceptable model fit, we concluded that measurement invariance holds for all classes.

**TABLE 4:** Measurement invariance assessing equivalence of AARC-10 SF model across groups defined by age, sex, marital status, education group, and employment.

Models	RMSEA (90% CI)	CFI	SRMR
Age group			
Model 1: Configural invariance	0.07 (0.06; 0.08)	0.95	0.06
Model 2: Metric invariance	0.07 (0.06; 0.08)	0.94	0.07
Model 3: Strong invariance	0.07 (0.07; 0.08)	0.92	0.07
Sex			
Model 1: Configural invariance	0.07 (0.06; 0.08)	0.95	0.06
Model 2: Metric invariance	0.07 (0.06; 0.07)	0.95	0.06
Model 3: Strong invariance	0.07 (0.06; 0.08)	0.94	0.06
Marital status			
Model 1: Configural invariance	0.07 (0.06; 0.08)	0.95	0.06
Model 2: Metric invariance	0.06 (0.06; 0.07)	0.95	0.06
Model 3: Strong invariance	0.06 (0.06; 0.07)	0.95	0.06
Education group			
Model 1: Configural invariance	0.07 (0.06; 0.08)	0.95	0.06
Model 2: Metric invariance	0.07 (0.06; 0.07)	0.95	0.06
Model 3: Strong invariance	0.07 (0.06; 0.07)	0.94	0.06
Current employment			
Model 1: Configural invariance	0.07 (0.06; 0.08)	0.95	0.06
Model 2: Metric invariance	0.07 (0.06; 0.08)	0.94	0.06
Model 3: Strong invariance	0.07 (0.07; 0.08)	0.93	0.07

For the model with additional cross-loading, the measurement invariance was also established for all these classes (age group, sex, marital status, education group and current employment).

### Regression analyses

Regression analyses revealed that higher scores of AARC gains were associated with younger age and being female. The model predicted lower AARC losses scores for participants with university education, currently employed and if the participant was married or in a civil partnership or co-habiting (Table 5).

**TABLE 5:** Regression analyses with demographic variables as predictors of AARC gains and losses.

	Demographic variables as predictors of AARC gains: Simple regressions			Demographic variables as predictors of AARC gains: Multiple regression		
		
Variable	Coeff (95% CI)	*P*-value	Standardized Coeff.	Coeff (95% CI)	*P*-value	Standardized Coeff.
Age	−0.03 (−0.05; −0.01)	0.008	−0.07	−0.03 (−0.06; 0.00)	0.094	−0.06
Female	1.31 (0.88; 1.74)	<0.0001	0.15	1.21 (0.76; 1.65)	<0.0001	0.14
Marital status						
Widowed/separated/divorced/single	[Reference]	–	–	–	-	–
Married/civil partnership/co-habiting	−0.36 (−0.74; 0.03)	0.072	−0.05	−0.27 (−0.66; 0.12)	0.177	−0.04
Higher education	0.01 (−0.40; 0.42)	0.967	0.00	−0.04 (−0.45; 0.37)	0.844	−0.01
Currently employed	0.21 (−0.14; 0.56)	0.250	0.03	−0.12 (−0.58; 0.34)	0.612	−0.02
Total R^2^				0.03		
Adjusted R^2^				0.02		

	**Demographic variables as predictors of AARC losses: Simple regressions**			**Demographic variables as predictors of AARC losses: Multiple regression**		
		
**Variable**	**Coeff (95% CI)**	***P*-value**	**Standardized Coeff.**	**Coeff (95% CI)**	***P*-value**	**Standardized Coeff.**

Age	0.04 (0.02; 0.07)	<0.0001	0.10	0.00 (−0.03; 0.03)	0.818	0.01
Female	−0.36 (−0.78; 0.05)	0.089	−0.04	−0.34 (−0.76; 0.09)	0.121	−0.04
Marital status						
Widowed/separated/divorced/single	[Reference]	–	–	–	–	–
Married/civil partnership/co-habiting	−0.56 (−0.93; −0.19)	0.003	−0.08	−0.48 (−0.86; −0.11)	0.012	−0.07
Higher education	−0.57 (−0.97; −0.17)	0.005	−0.07	−0.49 (−0.88; −0.10)	0.015	−0.06
Currently employed	−0.94 (−1.27; −0.61)	<0.0001	−0.14	−0.82 (−1.26; −0.38)	<0.001	−0.12
Total R^2^				0.03		
Adjusted R^2^				0.03		

Regression analyses were repeated for the estimated factor scores using the model with the cross-loading as the outcome variables, and similar estimates were obtained; all the estimated effects were of similar magnitude, and the same variables appeared to be statistically significant.

### Correlation between gains and losses with cognitive functioning

All the cognitive test scores were modestly but statistically significantly correlated. All the test scores except for Trail-making B were positively correlated. The highest absolute value correlation was between Trail-making B and Grammatical Reasoning scores (R = −0.42, *P*-value < 0.0001). Gains and losses were differentially correlated with cognitive functioning (Table 6). Higher scores on AARC losses showed either negligible or small associations with poorer performance in almost all the cognitive tasks examined except from scores on the Stroop test. On the other hand, higher AARC gains were not significantly correlated with most of the cognitive tasks, except for poorer performance on verbal reasoning.

**TABLE 6:** Correlations between AARC-10 SF gains and losses and cognitive functioning (*R*, 95%CI, *P*-value).

Domain	Digit span	Paired associate learning	Grammatical reasoning	Self-ordered search	Switching stroop test	Trail-making B
AARC gains	−0.03 (−0.08; 0.02) *p* = 0.284	−0.03 (−0.08; 0.03) *p* = 0.306	−0.12 (−0.18; −0.07) *p* < 0.001	−0.05 (−0.11; 0.00) *p* = 0.057	−0.04 (−0.10; 0.01) *p* = 0.117	0.04 (−0.02; 0.09) *p* = 0.203
AARC losses	−0.06 (−0.12; −0.01) *p* = 0.021	−0.05 (−0.11; 0.00) *p* = 0.058	−0.09 (−0.14; −0.03) *p* = 0.002	−0.03 (−0.08; 0.03) *p* = 0.288	−0.10 (−0.16; −0.05) *p* < 0.001	0.09 (0.03; 0.14) *p* = 0.002

## Discussion

In order to confirm the two-factor structure (one factor for each of AARC gains and AARC losses) and the internal consistency of the Norwegian translation of the AARC-10 SF, and to further explore whether the AARC concept is interpreted consistently in the same way across different populations groups, we tested reliability for the gains and losses subscales of the Norwegian translation of the AARC-10 SF. We also explored whether the demographic characteristics age, sex, education level, marital status, and work status predict AARC gains and AARC losses.

We confirmed the two-factor structure (AARC gains and AARC losses) of the AARC-10 SF. The associations between the AARC items and the factors were all statistically significant and reasonably strong. This implies a consistency of the AARC, that allows cross-country comparison, with for example, the UK version of the PROTECT study, the German Socio-Economic Panel—Innovation Sample, the National Study of Daily Experiences, and the Longitudinal Study Aging Amsterdam in the Netherlands. Items assessing AARC gains capture different age-related changes from items assessing age-related losses. For instance, in the cognitive domain AARC losses capture declining mental capacity, whereas AARC gains capture increased experience and knowledge to evaluate things and people. Hence, individuals reporting low levels of AARC gains do not necessarily report high levels of AARC losses. In support of this reasoning, a study using latent profile analyses (19) showed that while some middle-aged and older individuals report concurrent high levels of AARC losses and low levels of AARC gains, others report both high levels of AARC losses and high levels of AARC gains. Moreover, other empirical studies (5, 36) have shown that levels of AARC gains are only minimally correlated to levels of AARC losses, suggesting that perceived gains are fairly independent from perceived losses. Another interesting finding was that there were statistically significant correlations between the “… *I pay more attention to my health*”—question, which captures perceived gains in the AARC physical health domain, and all the items attributed to AARC losses (*R* = 0.2–0.3, Table 3). It may be that while growing older, people pay more attention to their health for different reasons. Some people may care more about their health and health-related behaviors driven by the desire to maintain physical fitness as long as possible, whereas other people need to care more about their health in order to cure or manage the health conditions they may have (15, 37). Hence, caring about one’s own health may be related both to positive and negative age-related changes.

We found that the AARC gains and AARC losses factors and each item of the AARC-10 SF were consistently interpreted in the same way by men and women, people who obtained a university degree and those who did not, across different age groups, and employed and retired or unemployed individuals. This finding enables future studies to make accurate comparisons of AARC scores in these subpopulations of individuals. This is important as both predictors and outcomes of AARC gains and losses may differ across different subpopulations of individuals.

There are five AARC gains and five AARC losses in different AARC behavioral domains such as lifestyle/engagement, cognitive functioning, health and physical functioning, socio-cognitive and socio-emotional functioning and interpersonal relationships. As the associations between levels of AARC gains and AARC losses and demographic characteristics seem to vary across cohorts and countries, it is interesting to look further into these aspects in a Norwegian context. In our study, we found that higher scores of AARC gains were associated with younger age and being female. In addition, participants having a university education, currently being employed, being married, in a civil partnership or living together with someone reported lower AARC losses scores. These findings are consistent with previous evidence exploring AARC in middle-aged and older people living in the UK (18). Hence, there seem to be no cultural differences between the PROTECT study cohorts in the UK and Norway in the associations of AARC gains and losses with demographic variables. To what degree this can be generalized to the countries’ population aged 50+ has not been explored, but the finding is in line with existing evidence on sex differences in AARC and subjective wellbeing (15, 17).

Furthermore, scores on AARC gains and losses were differentially correlated with cognitive functioning. In line with previous evidence, perceived age-related losses seem to be only minimally informative of the objective concurrent cognitive performance of middle-aged and older people (5, 38). Many variables, including individual beliefs about aging and mood, may explain why perceived age-related losses show small associations with objective cognition (26). Interestingly, our finding that poorer performance on the verbal reasoning task, is related to higher perceived age-related gains, confirms previous findings (5) and highlights the need for further research unraveling factors underpinning the counterintuitive association of more AARC gains and poorer cognition. A possible explanation may be that higher levels of AARC losses are often accompanied by higher levels of AARC gains, hence those people with poorer cognition and higher levels of AARC losses also report higher levels of AARC gains (18).

For the Norwegian health authorities, these findings are of great value as the AARC measure can map positive age-related changes (AARC gains) and lower levels of awareness of negative age-related changes (AARC losses). These dimensions are paramount in increasing the knowledge pertaining to active and healthy aging—e.g., adapting to age-related challenges, being physically active and engaged in leisure activities, as more AARC gains and fewer AARC losses are associated with better concurrent cognitive, physical, and mental health among middle-aged and older individuals (4–6), and also predict better future physical and mental health and even less mortality risk (7–9). In sum, asking individuals about the gains and losses they experience in several domains of their lives may help to identify those who are struggling the most while getting older.

In terms of study limitations, there might be a risk that the study sample is biased, not being representative of the Norwegian population as a whole since the recruitment had a strategic approach. Study participants were recruited from different sources, including a healthy cohort of hospital clinical studies, senior citizen associations, and a social media advertising campaign.

## Conclusion

The Norwegian translation of the AARC-10 SF was validated. It could be useful in clinical and counseling settings to identify those people who, because they report few AARC gains and many AARC losses, may benefit from intervention programs promoting adaptation to age-related changes and/or engagement in health-promoting behaviors. Specifically, higher AARC losses scores are associated with being male, having lower education, being professionally inactive and living alone. Furthermore, AARC can potentially contribute significantly to the Governments plans for an aging society, such as “A full life- all your life- a quality reform for older persons” (39) and, in general age-friendly societies.

## Data availability statement

The raw data supporting the conclusions of this article will be made available by the authors, without undue reservation.

## Ethics statement

The studies involving human participants were reviewed and approved by the Regional Committee for Medical and Health Research Ethics, West (Ref #2019/478). The patients/participants provided their written informed consent to participate in this study.

## Author contributions

SS conducted the literature review. JA and AU processed the data. AU conducted the statistical analysis and the interpretation of results with input from SS and MG. The final and re-submitted manuscript was critically revised by IT and subsequently by the whole group before submission. All authors jointly contributed in this process of the writing and revision of the manuscript, and contributed to devising the study objectives, and contributed to the article and approved the submitted version.

## Footnotes

1
https://www.protect-norge.no/
